# Heparin Anticoagulant Therapy and Its Monitoring

**DOI:** 10.3390/biom16030425

**Published:** 2026-03-13

**Authors:** Benjamin Reardon, Leonardo Pasalic, Giuseppe Lippi, Emmanuel J. Favaloro

**Affiliations:** 1 Joint Medical Program, School of Medicine and Public Health, University of Newcastle, Callaghan, NSW 2145, Australia; benjamin.reardon@health.nsw.gov.au; 2 Haematology Department, Calvary Mater Hospital Newcastle, Waratah, NSW 2298, Australia; 3 Haematology Department, Institute of Clinical Pathology and Medical Research (ICPMR), NSW Health Pathology, Westmead Hospital, Westmead, NSW 2145, Australia; leonardo.pasalic@health.nsw.gov.au; 4 Westmead Clinical School, University of Sydney, Westmead, NSW 2145, Australia; 5 Sydney Centres for Thrombosis and Haemostasis, Research and Education Network (REN) and Institute of Clinical Pathology and Medical Research (ICPMR), Westmead Hospital, Westmead, NSW 2145, Australia; 6 Section of Clinical Biochemistry, University of Verona, 37129 Verona, Italy; giuseppe.lippi@univr.it; 7 School of Dentistry and Medical Sciences, Faculty of Science and Health, Charles Sturt University, Wagga Wagga, NSW 2650, Australia; 8 School of Medical Sciences, Faculty of Medicine and Health, University of Sydney, Westmead Hospital, Westmead, NSW 2145, Australia

**Keywords:** heparin, enoxaparin, LMWH, anticoagulation, thrombosis

## Abstract

Heparin remains a foundational parenteral anticoagulant across both acute and chronic care settings. This narrative review summarizes clinical indications and dosing of unfractionated (UFH) and low-molecular-weight heparin (LMWH). It also details laboratory monitoring using activated partial thromboplastin (APTT), anti-factor Xa (anti-Xa), activated clotting time (ACT) and viscoelastic testing (VET), including common pitfalls and interferences. We provide considerations for specific populations as well as complications including heparin resistance, heparin-induced thrombocytopenia (HIT) and heparin reversal strategies. Future research directions include harmonization of therapeutic ranges, mitigation of assay interference and prospective evaluation on monitoring, particular in extracorporeal membrane oxygenation (ECMO), pregnancy and cardiac surgical settings.

## 1. Introduction

Heparin was first isolated in the early 20th century; despite multiple changes and developments within antithrombotic and anticoagulant therapy, this anticoagulant retains a pivotal role due to its reliability, rapid onset, titratability, reversibility, and monitoring capability [1]. Heparin was originally described by Maurice Doyon in 1910 [2], copurified as an anticoagulant in 1916 [3], and later developed as a clinically used anticoagulant in the 1930s by Charles Best in Canada and Erik Jorpes in Sweden [4]. Unfractionated heparin (UFH) acts by accelerating antithrombin-mediated inhibition of thrombin (factor IIa) and factor Xa [5]. Low-molecular-weight heparin (LMWH), generated by the depolymerization of UFH, exerts a proportionally greater effect on factor Xa, owing to shorter chain length, whilst retaining some of its anti-IIa activity [6]. Pharmacokinetic differences have significant implications: UFH binds to plasma proteins and endothelial cells, resulting in nonlinear, highly variable kinetics among treated patients, often reflected by ~30% bioavailability at therapeutic dosing with a dose-related half-life of ~0.5–1.5 h, thus requiring frequent laboratory monitoring and dose adjustment at therapeutic administration [6]. Conversely, LMWH has less non-specific binding, resulting in more predictable bioavailability and clearance, with ~90–100% bioavailability for enoxaparin, ~80–90% for dalteparin [6] and longer elimination half-life of about ~3–7 h [7], thus enabling fixed or weight-adjusted dosing with the need for monitoring only in limited specific scenarios [8,9,10]. Examples of the most commonly used LMWHs include dalteparin (Fragmin), enoxaparin (Lovenox, Clexane) and tinzaparin (Innohep) [10]. Beyond anticoagulation, heparin exhibits anti-inflammatory and immunomodulatory properties, including interactions with neutrophil extracellular traps (NETs) and complement, though translational clinical impact remains context dependent [11,12]. Accordingly, this review aims to map contemporary evidence on UFH and LMWH pharmacology, complications including HIT and heparin resistance, laboratory monitoring principles and their limitations, and to identify gaps where assay standardization, outcome-linked therapeutic ranges, and clinically accepted applications have limited evidence.

## 2. Clinical Use of Heparin

### 2.1. Indications

UFH and LMWH form the backbone of prophylactic and therapeutic anticoagulation and are preferred in many inpatient and some outpatient settings. Both UFH and LMWH can be administered as prophylaxis of venous thromboembolism (VTE) for medium and high-risk patients (including surgical, medical, and orthopedic patients), as well as at therapeutic dosing in management of deep vein thrombosis (DVT), pulmonary embolism (PE) and acute coronary syndrome (ACS) [13,14,15]. Therapeutic dosing of UFH should be considered a high-risk medicine, given its narrow therapeutic window, and that overdosing or underdosing can result in significant adverse patient outcomes in hospitalized patients [16]. UFH is increasingly being replaced as the first-line anticoagulant by LMWH and direct oral anticoagulants (DOACs), especially in patients with atrial fibrillation (AF) for primary or secondary stroke prevention who do not require bridging therapy [17]. UFH is favored when rapid titration, short half-life, reversibility, or procedural control is essential, whereas LMWH is preferred for medically stable VTE and malignancy-associated thrombosis owing to more predictable pharmacodynamics and pharmacokinetics [18]. A comparison of UFH and LMWH is summarized in Table 1.

### 2.2. Dosing Approaches

Dosing for UFH and LMWH should be divided into prophylactic, therapeutic, and intermediate-dose groups. Prophylactic dosing for UFH is weight-adjusted or fixed dose (2500 IU or 5000 IU) based on twice- or three-times-daily dosing [19], and LMWH is either weight-based (0.5 mg/kg once daily) or fixed-dose regimens (40 mg once daily) [10]. For therapeutic anticoagulation, UFH practice typically involves weight-based nomograms—commonly an initial intravenous bolus of ~80 units/kg followed by 18 units/kg/h—with titration to a single monitoring modality based on activated partial thromboplastin time (APTT) or anti-Xa, which will be discussed later [20]. Therapeutic LMWH is administered subcutaneously using fixed or weight-based regimen (1 mg/kg administered twice daily, or 1.5 mg/kg as a single daily dose). Routine laboratory monitoring for LMWH is unnecessary in most adults, but should be considered in pregnancy, extremes of body weight (<60 kg and >150 kg or BMI > 40 kg/m^2^), and significant renal impairment [9,10]. There is a lack of consensus regarding dose capping, or setting a maximum dose regardless of total weight, which carries a theoretical risk of underdosing and higher risk of VTE recurrence. In a systemic review by Liu et al. (2023), therapeutic LMWH dosing in obese adults (defined as BMI > 30 kg/m^2^) found that dose capping resulted in similar VTE recurrence rates (odds ratio (OR): 0.86, 95% confidence interval (CI): 0.11–6.84, *p* = 0.89), but a lower incidence of bleeding events (OR: 0.30, 95% CI: 0.10–0.89, *p* = 0.03) [21]. This was not seen in LMWH prophylaxis in obese adults, whereby higher-dose LMWH had a lower incidence of VTE compared with standard-dose regimens (OR: 0.47, 95% CI: 0.27–0.82, *p* = 0.007) and similar incidence of bleeding events (OR: 0.86, 95% CI: 0.69–1.08, *p* = 0.020). Intermediate dose of UFH and LMWH has been explored in some patient populations, including coronavirus disease 2019 (COVID-19), but can be considered in patients with DVT or PE or ACS with high bleeding risk or recent/active bleeding [22].

### 2.3. Complications

Bleeding is the main risk with both UFH and LMWH therapy. Overdose of UFH or LMWH may result in spontaneous or worsening of provoked hemorrhage, which may be life-threatening [10]. Clinical presentations include severe bleeding, compartment syndrome, post-surgical bleeding (including intracranial hemorrhage, hemothorax, retroperitoneal bleeding or intra-abdominal bleeding), disseminated intravascular coagulation (DIC) and multi-organ failure [23]. Overdose or active bleeding may require urgent reversal with protamine or other blood products, which is later discussed. Other complications are less common and include osteoporosis, spontaneous fractures, hypoaldosteronism, hypersensitivity reactions and heparin-induced thrombocytopenia (HIT), sometimes also presenting with thrombosis (HITT) [24,25,26,27,28], which are later discussed in detail. Dalteparin has warnings for its use in pregnant women and neonates due to the preservative benzyl alcohol, which may cause ‘gasping syndrome’, characterized by central nervous system (CNS) depression, gasping and metabolic acidosis in neonates [29,30], although there are preservative-free formulations now available.

### 2.4. Contraindications

Caution should be had for use of UFH or LMWH in patients with bleeding diathesis, uncontrolled arterial hypertension, recent gastrointestinal bleeding, and diabetic retinopathy, all of which enhance the hemorrhage risk. Caution should also be used in patients with renal impairment, with most guidelines suggesting that LMWH is contraindicated when creatinine clearance (CrCl) falls to <10–15 mL/min [31,32,33].

## 3. Laboratory Monitoring Modalities

### 3.1. Activated Partial Thromboplastin Time (APTT)

**Figure 1:**
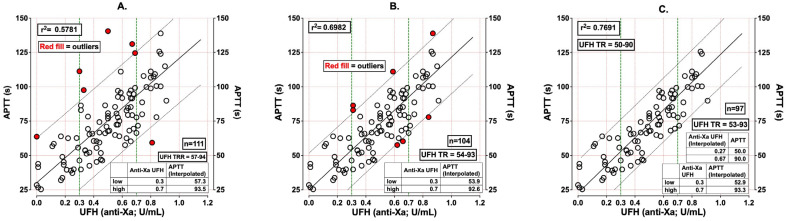
The process of generating an APTT unfractionated heparin (UFH) therapeutic reference range (TRR). (**A**). The initial data set (n = 111) comprising samples from patients on UFH, preferably with normal prothrombin time (PT) to help exclude capture of hemostasis confounders such as liver disease or factor deficiency. Regression analysis identifies the line of best fit plus 95% confidence intervals. The UFH TRR is interpolated from the anti-Xa range of 0.3–0.7 U/mL, which is 57–94 s in this example. Outlier data points are identified by the red-filled symbols. High-APTT outliers may comprise samples from patients with factor deficiency or lupus anticoagulant. Low-APTT outliers may comprise samples from patients with high factor levels (e.g., fibrinogen, FVIII). These outlier points are removed and the analysis is repeated. (**B**). After removal of outliers, with n = 104, the UFH TRR does not change markedly (now 54–93 s), and the r^2^ improves slightly. A second round of outlier points could be identified and were also removed. (**C**). After removal of these additional outliers, with n = 97, the UFH TRR does not change appreciably (now 53–93 s), and the r^2^ again improves slightly. The UFH TRR of 53–93 s may be difficult to memorize, and may also suggest an accuracy not present in these evaluations. Therefore, the UFH TRR is adjusted to 50–90 s. Interpolation of these values gives an anti-Xa range of 0.27–0.67 U/mL UFH, which, with rounding, is 0.3–0.7 U/mL, and thus represents an acceptable UFH TRR.

APTT testing remains widely available and inexpensive, reflecting global intrinsic and common pathway activity [34]. There is substantial reagent and analyzer variability necessitating local calibration of therapeutic range to anti-Xa ~0.3–0.7 IU/mL [35] (Figure 1). An APTT ratio of 2.0–3.5× the laboratory control is often associated with therapeutic UFH, rather than the historical 1.5–2.5× reference range [5,8]. For laboratories unable to perform anti-Xa, the use of less-responsive APTT reagents with a ratio target of ~2.0–3.5× has been proposed as a reasonable surrogate to approximate therapeutic anti-Xa levels, though this remains inferior to assay-specific calibration and should be locally validated [8,36].

### 3.2. Anti-Factor Xa (Anti-Xa)

Anti-Xa represents a functional assay that measures chromogen generation proportional to the level of residual factor Xa after neutralization by UFH–antithrombin (AT) complex, and is inversely proportional to the heparin concentration [37]. It should be noted that this assay measures only AT-catalyzed inhibition of factor Xa and does not account for the anti-IIa activity of UFH [38]. Unlike APTT, anti-Xa assays directly quantify heparin–AT inhibition of factor Xa, providing a biochemical target of ~0.3–0.7 IU/mL for UFH, and enabling peak monitoring for LMWH when indicated [38]. A critical caveat is interference from FXa-inhibiting DOACs, which greatly elevate heparin-calibrated anti-Xa values, mandating drug-specific assays or pre-analytical DOAC removal approaches in clinical contexts [39,40]. Beyond ex vivo neutralization, other strategies to remove DOAC effect include timed sampling at the expected trough or after an adequate washout, recognizing that practical windows may exceed 24–48 h because of pharmacokinetic variability, impaired renal function and comedications [40,41]. Anti-Xa monitoring is preferred where a patient’s APTT is abnormal at baseline, which may be due to the presence of a lupus anticoagulant (LA), such as in antiphospholipid syndrome (APS), or in inherited or acquired factor deficiencies (VIII, IX, XI and XII), or in patients not responding to heparin based on APTT [38].

### 3.3. Activated Clotting Time (ACT)

ACT is a point-of-care (POC) functional whole-blood test commonly used in hospital settings, and is the standard of care for cardiac procedures with large UFH dose administration, as well as non-cardiac vascular surgery. ACT provides largely intraoperative coagulation monitoring [42,43,44,45,46], but may be affected by several patient-related factors (including hypothermia, hemodilution, platelet count, factor deficiencies), all of which may affect heparin under- or overdosing as well as bleeding risk [47]. Multidisciplinary guidelines recommend maintaining ACT at or above 480 s, with acceptance of ≥400 s for devices that employ maximal activation, although there is significant variability between devices and institutional protocols [43,48,49].

### 3.4. Viscoelastic Testing (VET)

Viscoelastic testing (TEG/ROTEM) provides rapid, whole-blood assessment of clot initiation, propagation, strength, and lysis, integrating platelet and fibrin contributions [50]. Heparin effects can be explored via heparinase channels, or TEG-H; or by comparing INTEM with HEPTEM using ROTEM. While VET is invaluable for bleeding management algorithms in cardiac surgery and critical care, its diagnostic performance for detecting residual heparin after protamine administration is variable, with randomized and observational work showing no clear superiority over ACT and susceptibility to confounding by protamine and heparinase [12,51,52].

### 3.5. Pre-Analytical Considerations, Interference and Comparison

Accurate interpretation of APTT for heparin monitoring is limited by a number of pre-analytical variables, including heparin contamination and inappropriate sample collection leading to spurious activation of blood coagulation [53], as well as patient-specific factors such as inflammation, high fibrinogen and factor VIII, and the presence of LA, all of which can prolong the clotting time of the assay independent of heparin [54,55]. In particular, if an LA-sensitive APTT reagent is used, the presence of LA can prolong phospholipid-dependent clotting times, complicating APTT-based titration [34]. In this circumstance anti-Xa monitoring is preferable [38]. A theoretical alternative is the use of an LA-insensitive APTT reagent.

As mentioned, common interferences for both APTT and anti-Xa monitoring include concurrent or overlapping anti-Xa DOAC use. Laboratories should provide DOAC-neutralization options (e.g., DOAC-Stop or DOAC-Remove; [39]) or include qualifying statements in their reporting to alert clinicians to potential interactions [40]. Activated-charcoal-based adsorbents including DOAC-Stop and DOAC-Remove remove DOACs ex vivo from citrated plasma and have been validated by mass spectrometry and functional assays to markedly reduce residual apixaban, rivaroxaban, and dabigatran to concentrations unlikely to affect most coagulation tests [56]. Additional pre-analytical factors that may influence both APTT and anti-Xa assays include incorrect timing (non-steady-state sampling or non-peak draws), under-filled citrate tubes, and delayed processing or transcription errors [55].

Viscoelastic testing as a measure of whole-blood coagulation can have interferences from fibrinogen, platelets, and factor compartments affecting interpretability [50]; however, interpretative algorithims and heparin-specific cartridges largely mitigate incorrect interpretation of these, provided there is adequate user-familiarity with the method used. Viscoelastic testing may be an increasingly used adjunct post cardiac surgery, since it already has a growing evidence base including the benefit of reduced blood product use [57,58,59,60,61,62,63,64,65,66,67]. Viscoelastic assays require strict pre-analytical control because whole-blood samples must be tested promptly—ideally within minutes—as delays reduce clot amplitude and distort kinetic parameters [50,68]. Hematocrit, platelet count, and residual heparin markedly alter viscoelastic traces, and inappropriate use of heparinase channels or activators can misrepresent the heparin effect [68,69].

Table 2 summarizes the key differences, limitations and interferences of different assays in UFH and LMWH monitoring.

There have been some reports of transitioning from APTT to anti-Xa monitoring with quicker attainment of therapeutic values, fewer rate changes, and operational efficiencies, although there are no randomized prospective data to guide this [9,70]. Discordance between APTT and anti-Xa is not uncommon, commonly due to elevations in factor VIII/fibrinogen or reductions in factor levels/presence of LA that shorten or prolong APTT disproportionally [70] (Figure 1).

**Table 2:** A comparison of heparin monitoring.

Assay	Turn Around Time	Principle	Therapeutic Targets	Advantages	Limitations and Interferences	Practical Considerations
Anti-factor Xa (chromogenic)	Laboratory dependent (~30 min to 4 h)	Measures FXa inhibition proportional to heparin–AT activity (chromogenic substrate).	UFH: anti-Xa ~0.3–0.7 IU/mL; LMWH: peak 3–5 post-dose [9,71].	Direct activity measure; avoids APTT reagent variability; aligns UFH titration to biochemical target.	Interferences: FXa DOACs cause falsely high anti-Xa; requires drug-specific assays or DOAC removal. Availability: not available in all sites managing heparin therapy.	Preferred for UFH when APTT correlation poor; LMWH monitoring in pregnancy; extremes of weight; renal impairment.
APTT	Laboratory dependent (~30–60 min)	Global intrinsic/common pathway clot-based time; indirectly reflects UFH via AT-mediated IIa/Xa inhibition.	Normally: reagent-specific; UFH therapeutic: ratio matching anti-Xa 0.3–0.7 IU/mL [35]; often ~2.0–3.5× control [5,8].	Widely available; inexpensive; feasible when anti-Xa invalidated by recent anti-FXa-DOAC exposure.	Marked inter-reagent variability; prolonged by lupus anticoagulant, factor deficiencies; reduced by acute-phase reactants; DOAC interference.	Use lab-specific therapeutic ranges correlated to anti-Xa [35,72] (Figure 1); avoid sampling from heparinized lines. Consider LA-insensitive reagent.
ACT	POC	Rapid POC whole-blood clotting time with strong contact activation; practical at high UFH levels.	Baseline: device-specific; CPB: ≥480 s (≥400 s acceptable with maximally activated systems) [51].	Immediate POC feedback; essential for high-dose UFH in CPB; integral to protamine titration.	Non-standardized; affected by hypothermia, hemodilution, platelet dysfunction, factor deficiency	Use device-specific targets and quality control; consider adjunct anti-Xa when ACT discordant.
Viscoelastic Testing (TEG/ROTEM)	POC	POC whole-blood viscoelastic assay; heparin effect assessed via heparinase channels (TEG-H; or INTEM vs. HEPTEM for ROTEM).	No universal numeric target; used qualitatively; adjunct for protamine titration and coagulopathy assessment [12].	Global hemostatic view including platelets and fibrin; useful in bleeding algorithms for cardiac surgery/ECMO [48,52,73].	Limited sensitivity for residual UFH; confounded by protamine and heparinase; poor correlation with anti-Xa at low levels of UFH [74].	Adjunct only; anti-Xa remains preferred for UFH [74].

Abbreviations: APTT, activated partial thromboplastin time; ACT, activated clotted time; AT, antithrombin; DOAC, direct oral anticoagulant; ECMO, extracorporeal membrane oxygenation; FXa, factor Xa; LA, lupus anticoagulant; LMWH, low-molecular-weight heparin; POC, point of care; TEG-H, thromboelastography-heparin; UFH, unfractionated heparin.

## 4. LMWH and Special Populations

LMWH use has expanded rapidly and has an emerging research base in particular populations. Of note, routine anti-Xa monitoring is not recommended in ‘standard’ patient groups because therapeutic ranges correlated to clinical outcomes are lacking [10,75,76]. Monitoring may be considered in special populations, including pregnancy, renal impairment, extracorporeal membrane oxygenation (ECMO), and extremes of body weight, with standardized peak timing (~4 h post-dose at steady state) and explicit targets for prophylactic versus treatment dosing [9,75,76]. Real-world studies show that many anti-Xa levels are drawn outside the optimal window and that out-of-range values do not consistently drive appropriate dose modifications [77,78].

### 4.1. Pregnancy

LMWH is the preferred anticoagulant in pregnancy because it does not cross the placenta, has predictable pharmacokinetics, and is associated with low rates of VTE recurrence and adverse events in observational cohorts and systematic reviews [79,80,81]. The guidelines of the American Society of Hematology strongly recommend LMWH over UFH for acute VTE in pregnancy, and also endorses antepartum prophylaxis for women with a history of unprovoked or hormone-associated VTE, with universal emphasis on postpartum prophylaxis if there has been a history of prior VTE [82].

Indications and dosing span prophylactic, intermediate, and therapeutic intensity, selected by baseline risk and clinical presentation, and are weight-based, generally taken from the weight at the beginning of the pregnancy or at the time of the VTE event [82]. Prophylactic LMWH is used for moderate-risk patients (e.g., prior provoked VTE with additional risk factors or certain thrombophilias) and is commonly delivered as fixed low-dose regimens such as enoxaparin 40 mg once daily [82]. The combination of prophylactic-dose LMWH and aspirin in pregnant women with APS has been shown to improve the live birth rate [82,83,84]. Intermediate-dose LMWH (e.g., enoxaparin 40 mg twice daily) is considered for higher-risk patients (e.g., previous unprovoked or estrogen-related VTE), although the Highlow randomized trial found no reduction in recurrent VTE with intermediate- versus low-dose LMWH during pregnancy and six weeks postpartum [84,85,86]. Therapeutic dosing—typically weight-adjusted (e.g., enoxaparin 1 mg/kg twice daily)—is indicated for current VTE, multiple prior VTEs, or high-risk thrombophilia. Nevertheless, UFH may be preferred in select settings such as severe renal impairment or when rapid reversal is anticipated [82,87]. Peripartum management commonly includes stopping anticoagulation 24 h before planned delivery or neuraxial anesthesia. Of note, retrospective data show no significant difference in postpartum hemorrhage with or without conversion from LWMH to UFH peripartum, although some centers continue this practice to facilitate anesthesia management [88,89].

Monitoring for LMWH in pregnacy with anti-Xa activity is controversial and largely based on expert consensus [90,91]. The 2019 European Society of Cardiology [92] and American Society of Hematology 2018 guidelines [82] recommend reserving anti-Xa monitoring for specific high-risk circumstances such as recurrent VTE, renal impairment or extremes of body weight. In high-risk scenarios, anti-Xa monitoring may be performed every 2–4 weeks to maintain trough levels above 0.1 IU/mL and peak levels (4 h post-injection) between 0.5 and 1.0 IU/mL [93].

### 4.2. Renal Impairment

In patients with renal impairment, the choice between UFH and LMWHs hinges on the differences in elimination and accumulation risk. UFH is largely cleared by reticuloendothelial mechanisms and can be used at standard therapeutic infusion doses across creatinine-clearance ranges [9,94]. In contrast, LMWHs are partly eliminated by the kidneys and can accumulate in patients with lower CrCl, increasing the bleeding risk; most guidelines suggest dose reduction when CrCl < 30 mL/min and consideration of switching to UFH in advanced chronic kidney disease or dialysis-dependent patients [95,96,97]. Enoxaparin therapeutic dosing is typically 1 mg/kg once daily when CrCl < 30 mL/min (versus 1 mg/kg twice daily if CrCl ≥ 30 mL/min), while other LMWHs (e.g., dalteparin, tinzaparin) have variable dose recommendations [32,33,98].

Dose monitoring and drug titration is recommended for patients with renal impairment who require any dose modification or when CrCl < 30 mL/min, with goal peak ranges reported as anti-Xa ~0.5–1.0 IU/mL for twice-daily regimens and trough measurements helping to detect accumulation [78]. After an initial steady-state peak, repeating anti-Xa is only recommended by most guidelines only when clinical status changes (e.g., bleeding, dose changes, or renal function shifts) [78,99]. For enoxaparin in advanced chroncic kideny disease (CKD), prospective and retrospective data show substantial proportions outside the target after the first check—prompting some centers to re-check within 5–7 days or after any dose/renal change, rather than at fixed frequent intervals [99,100]. In severe renal impairment, however, UFH remains preferred over LMWH [94,95].

### 4.3. ECMO

ECMO is a life support modality for adults and children with life-threatening cardiac and pulmonary failure, allowing temporary oxygenation and blood circulation [101,102]. ECMO exposes the blood compartment to extensive circuitry, leading to contact pathway activation, thrombin generation and consumption of antithrombotic proteins [103]. Often concurrently, critical illness leads to an increase in acute-phase reactants and inflammatory mediators [104,105], causing significant inter- and intra-patient variability in UFH response and assay discordance in these patients [106]. UFH remains recommended as a continuous infusion for all patients receiving ECMO therapy [48,106,107].

ACT has historically been more widely used for heparin monitoring for patients on ECMO [108,109]. Nevertheless, the International Society on Thrombosis and Haemostasis (ISTH) now recommends anti-Xa monitoring, aiming for a target between 0.3 and 0.5 U/mL [73]. Ranucci (2020) has described several issues with using anti-Xa alone in patients receiving ECMO, including interference from bilirubin and free hemoglobin, which can affect chromogenic assay reliability [109]. Alternatively, APTT and VET can be used adjunctively, although they are all susceptible to hypothermia, hemodilution, thrombocytopenia, and LA, as well as acute-phase reactants and assay/device-related factors [75,110,111,112]. For patients with VET, heparin inactivators are typically used, so that the anticoagulant effect can be separated from other factors [50,112]. HIT and heparin resistance have been commonly reported as complications in patients on ECMO, in whom bivalirudin or argatroban may be used, recognizing altered clearance in multi-organ dysfunction and the need for institution-specific protocols [113].

### 4.4. Extremes of Body Weight

In patients at the extremes of body weight, the pharmacokinetics of parenteral heparins shift in ways that effect efficacy and safety. This includes patients with low body weight (≤45–50 kg or BMI ≤ 18.5 kg/m^2^) or high body weight (>120 kg or BMI ≥ 40 kg/m^2^). Current guidelines overall recommend tailored dosing with monitoring [114].

Having a low body weight is associated with higher anti-Xa exposure for patients receiving LMWH. Observational studies of prophylaxis show that reduced-dose enoxaparin (e.g., 20–30 mg once daily) achieves similar VTE outcomes with less bleeding than standard 40 mg daily [115,116,117,118]. For patients with high body weight, LMWH is associated with lower anti-Xa exposure at fixed prophylactic doses [21] although a subsequent meta-analysis suggests that higher prophylactic LMWH doses reduce VTE without increasing bleeding in adult patients, while therapeutic dosing should remain weight-based (actual body weight) rather than capped [119]. A 2024 systematic review of therapeutic enoxaparin reported higher proportions of in-range anti-Xa with reduced weight-based dosing (~0.75–0.85 mg/kg) versus standard ≥ 0.95 mg/kg in patients ≥ 100 kg or BMI ≥ 40 kg/m^2^, with most bleeding events clustering at standard doses [120]. This, as well as a subsequent review, highlights the need for close anti-Xa monitoring in actual body weight-based dosing in this patient group [121].

For UFH, high body weight can alter the distribution volume of heparin responsiveness, as it is mainly limited to plasma with minimal distribution in adipose tissue [122,123]. Weight-based infusion nomograms are typically used; however, patients with high body weight (BMI ≥ 40 kg/m^2^) are more likely to exhibit supratherapeutic APTT values compared with non-obese patients when dosing is based on total body weight, highlighting the need for closer laboratory monitoring in this patient group [124,125,126]. In a retrospective audit, George et al. (2020) found that UFH dosing in obese adults (i.e., >150 kg) required lower U/kg/h than in patients < 100 kg (11.6 ± 4.2 vs. 16 ± 4.1, respectively) [127]. In another retrospective study by Nguyen et al. (2025), actual body weight compared with total body weight did not result in a higher likelihood of achieving a therapeutic initial APTT target with UFH (54.2% versus 41.9%, *p* = 0.238) [128]. When using anti-Xa–based heparin monitoring, Flanagan et al. (2025) found that patients with BMI ≥ 30 kg/m^2^ achieved therapeutic anti-Xa levels with significantly lower heparin dose (14 units/kg/h vs. 16 units/kg/h, *p* < 0.001) [129]. Patients in the obese group also had significantly more supratherapeutic anti-Xa levels within the first 24 h (50% vs. 33%, *p* < 0.0001) and throughout the total duration of UFH therapy (40% vs. 25%, *p* < 0.0001) without significant differences in bleeding events. A summary of dosing and monitoring strategies is given in Table 3.

### 4.5. Orthopedic Surgery

In orthopedic surgery, LMWH reduces symptomatic VTE compared with no prophylaxis and performs similarly to rivaroxaban in non-major procedures; trial and meta-analytic data suggest that initiation 6–24 h postoperatively balances bleeding and efficacy without clear superiority of one specific start time [130,131,132,133]. For patients requiring UFH at therapeutic dosing, low-intensity protocols may be utilized in high-risk patients or early postoperatively, which may include omission of bolus, lower APTT (50–60 s) or anti-Xa target (e.g., 0.1–0.3 U/mL) with tight monitoring and early reassessment.

## 5. Heparin Resistance

### 5.1. Definitions and Epidemiology

Heparin resistance is most commonly defined as minimal changes in APTT or ACT, or subtherapeutic anti-Xa levels, despite standard or increased UFH dosing (e.g., 35,000 or more units per day), although the threshold dose required is not well defined [16,91,134]. Clinically, heparin resistance refers to inadequate anticoagulation during procedures such as cardiopulmonary bypass despite escalating doses; nevertheless, laboratory-based definitions are increasingly used given variability in dosing scheduling [104,135]. In patients undergoing cardiac bypass, the definition of heparin resistance often used is the need for a dose of over 500 U/kg to achieve an ACT of 400 to 480 s [136]. The epidemiology of this condition varies widely by clinical context, with no universal prevalence due to differing definitions and patient populations, although it is commonly reported to range between 4 and 26% of adult cardiac surgery patients undergoing cardiopulmonary bypass (CPB) [137]. Clinicians may suspect heparin resistance when a therapeutic APTT is not reached within 24 h of heparin infusion. Consideration should be made for testing anti-Xa activity to determine efficacy, and if anti-Xa activity is subtherapeutic, an alternative anticoagulant may be considered [76,138].

### 5.2. Mechanisms and Management Approaches

Identification of the cause of heparin resistance depends on both clinical and laboratory information, considering that causes may be multifactorial. Because it carries a strong negative charge, heparin commonly binds to many proteins, leading to wide variability in patient response and dose requirements not seen with LMWHs [130]. UFH may bind to plasma proteins commonly elevated in critically ill patients, including acute-phase reactants, factor VIII and fibrinogen, chemokines and cytokines (including platelet factor 4 [PF4], tumor necrosis factor (TNF) and interleukin-8 (IL-8)), von Willebrand factor (VWF), adhesion molecules, microbial and nuclear proteins [104,134]. UFH may also bind to intravenous tubing and ECMO circuit components, potentially contributing to lower-than-expected APTT values by binding AT [139,140,141]. Other causes of heparin resistance include conditions characterized by reduced AT concentration, including congenital AT deficiency, liver disease, acute thrombosis, DIC or consumptive coagulopathy, surgery, hemodialysis or asparaginase use in patients with acute leukemia [142]. Notably, pre-analytic errors should always be a consideration when interpreting a result, particularly when samples are taken from intravenous (heparinized) lines.

When heparin resistance is suspected, a thorough understanding of the patient’s clinical context and status is essential to identify potential causes and determine the indications for current anticoagulation [104]. Initial review of pre-analytical factors should not be omitted, with the consideration of changing to anti-Xa when APTT interference is suspected. Measurement of AT activity can also assist with potential causes. There is no robust evidence that antithrombin concentrate is superior to fresh-frozen plasma (FFP), or that increasing overall heparin dosing is superior in these patients, given that AT levels may fall by approximately 30% after a patient first receives heparin, with levels gradually increasing again over time [104]. In congenital AT deficiency, there is some evidence supporting the use of anti-Xa-based anticoagulation and human recombinant AT for both prophylaxis [143] and as part of VTE therapy [144]. Andexanet alfa acts as a decoy factor Xa molecule, reversing the anticoagulant effects of direct factor Xa inhibitors as well as the indirect inhibitors including LMWH and UFH [145]. Andexanet alfa use in patients undergoing cardiac surgery to reverse apixaban or rivaroxaban often requires markedly increased doses of UFH to achieve target anticoagulation, highlighting another mechanism of heparin resistance [139]. A summary of causes of heparin resistance and management strategies are provided in Table 4.

## 6. Heparin-Induced Thrombocytopenia (HIT)

### 6.1. Clinical Probability Assessment (4T)

Heparin-induced thrombocytopenia (HIT) is an uncommon but potentially life-threatening complication of heparin therapy, especially if associated with thrombosis (HITT). HIT/HITT reflects an immune-mediated adverse reaction whereby heparin forms complexes with PF4, triggering IgG antibody binding to platelet Fc receptors, leading to platelet cross-linking and activation, thrombosis and thrombocytopenia [146]. Typically, if thrombocytopenia develops or the platelet count falls more than 30 to 50% below the patient’s own baseline over a 5-to-10-day period or 1–3 days with recent exposure, HIT may be suspected. Clinical assessment is essential for all patients with suspected HIT with the 4T score commonly used to assess pre-test probability [147]. The 4T score assists in laboratory and clinical-management-related decisions and assesses the degree of thrombocytopenia, the timing of onset after heparin exposure, thrombosis/other clinical sequelae, and other causes [148]. Although the 4T score can be used to predict the risk of HIT/HITT [149,150,151,152], HIT/HITT can be difficult to exclude or confirm based on clinical information alone, given the high frequency of thrombocytopenia in hospitalized patients [150]. There has been significant inter-observer variability in the use of the 4T score; however, a higher 4T score has remained significantly associated with positive HIT results [151]. In a prospective study, Larsen et al. (2024) found that a substantial number of patients with suspected HIT were misclassified based on the 4T score with a 13.5% false negative rate [152].

### 6.2. Laboratory Testing and Management Principles

Laboratory testing is essential for HIT/HITT diagnosis, but should not delay the institution of empiric therapy in suspected patients. Two types of assays exist: immunoassays and functional assays [28,153]. Immunoassays detect the presence of anti-PF4/heparin antibodies, while functional assays determine whether these antibodies activate platelets in the presence of heparin and are generally considered diagnostic [154]. Notably, only 10–50% of patients with positive immunoassays have platelet-activating antibodies on functional assays [117]. Because of their low positive predictive value, immunoassays alone cannot confirm or refute a diagnosis of HITT in a suspicious clinical scenario [155,156]. Performing a functional assay following a positive immunoassay is needed to minimize overdiagnosis and inappropriate treatment in patients without HITT [18]. Functional assays are generally considered as gold standards, i.e., ^14^C-serotonin release assay (^14^C-SRA) and heparin-induced platelet activation (HIPA). Nevertheless, these tests are limited in their availability, generally have long turnaround times and have a high cost involved [157].

Management of HIT entails cessation of all heparin and initiation of a non-heparin anticoagulant, although prophylactic or therapeutic dosing may vary depending on clinical context and pre-test probability [151]. DOACs may be considered after sufficient platelet recovery, although alternative non-heparin anticoagulants are preferred in the acute phase and depending on patient comorbidities [113,152].

## 7. Heparin Reversal

### Protamine Sulfate

Protamine, the only clinically available UFH reversal agent, binds UFH via electrostatic interactions to form an inactive complex, rapidly neutralizing anticoagulant activity within minutes [158]. Protamine was first described in the 20th century for its ability to neutralize heparin [159] but has also been shown to prolong clotting times when increasing amounts are added to whole blood [160], which was then later understood to exert an effect on platelet function, inhibition of factor V activation and down-regulation of thrombin generation [161]. Common dosing principles are weight- or dose-based titration targeting 1 mg protamine per ~100 U UFH given in the previous 2–3 h, with adjustments for elapsed time [162]. Population pharmacokinetics/pharmacodynamics modeling and perioperative studies of protamine suggest that lower protamine:heparin ratios (approximately 0.6–0.8:1) can achieve biochemical reversal while minimizing protamine excess, though operative contexts and monitoring strategies (ACT vs. anti-Xa) influence dose requirements [163]. In a meta-analysis on routine use of protamine following percutaneous coronary interventions, higher rates of hemostasis success (RR, 1.06; 95% CI [1.01–1.10]; *p* = 0.01) and shorter hospital length of stay (mean difference (MD), −0.46; 95% CI [−0.65, −0.26]; *p* < 0.01) were seen in patients who received complete heparin reversal compared with partial or none, highlighting its potential clinical benefit [164]. Post-cardiopulmonary bypass heparin rebound, or the reappearance of heparin anticoagulant activity following protamine reversal, is often demonstrated by increased thrombin time, anti-Xa activity, and ACT and protein-bound heparin levels, which can be mitigated by extended dose strategies [165]. Over-reliance of ACT alone can mask residual anti-Xa activity, supporting the need for multimodal monitoring when precise reversal is critical [161].

Adverse events range from transient hypotension and bradycardia to severe hypersensitivity reactions and anaphylaxis; thus, slow administration is recommended [106,166]. Risk factors include prior protamine exposure, use of protamine-containing insulins, prior vasectomy, rapid infusion, and high doses, though contemporary data suggest fish allergy per se confers low absolute risk and should not be considered a categorical contraindication when reversal is required [158]. Excess protamine may paradoxically impair coagulation via platelet effects and factor V interactions, and caution should thus be taken for prolonged infusions or repeated dosing [167]. Protamine only partially neutralizes LMWH, with more effective anti-IIa reversal and incomplete anti-Xa reversal, and its evidence remains limited [106]. Schroeder et al. (2011) reported that protamine neutralization depends on molecular size, with UFH fragments (mean molecular weight of 15,000 Da) neutralized but LMWH (5000 Da) not neutralized [168]. In vivo and ex vivo studies suggest tinzaparin (higher charge density) is more readily neutralized than enoxaparin, underscoring heterogeneity within the LMWH class [169]. Given only partial reversal, clinical judgment and supportive measures are key in LMWH-associated bleeding.

## 8. Conclusions and Future Directions

Both UFH and LMWH continue to play an essential role in acute and chronic anticoagulation due to their rapid onset, titratability, and established monitoring frameworks. Across clinical applications, dosing strategies remain highly context-dependent. Despite advances, monitoring practices remain heterogeneous; APTT, anti-Xa, ACT, and viscoelastic testing provide complementary insights, yet each remains an imperfect measure. Special populations—including pregnancy, renal impairment, ECMO, and extremes of body weight—demonstrate significant intra- and inter-patient variability, underscoring the need for individualized dosing and selective monitoring to mitigate bleeding and thrombotic risks. Future research priorities should include the following:

(1) Harmonization of therapeutic targets and laboratory monitoring, particularly the calibration of APTT to anti-Xa and the standardization of anti-Xa monitoring in LMWH therapy, where practice varies widely.
(2) Prospective studies comparing monitoring modalities including viscoelastic testing in high-risk settings such as ECMO.
(3) Clarifying the role of trough monitoring, defining optimal dosing at weight extremes, and larger prospective studies evaluating the role of anti-Xa monitoring during pregnancy, including in prophylactic and therapeutic VTE dosing.
(4) Strengthening evidence-based approaches to heparin resistance and HIT evaluation.

Beyond technical considerations of dosing and assay selection, the safe use of heparin is strongly influenced by systems-level aspects. Effective anticoagulation management relies on close collaboration between clinicians and laboratory services, especially regarding assay selection, sample timing, and the identification of analytical limitations and interferences. Standardized institutional pathways, supported by electronic prescribing, clear laboratory reporting and clinician education, may reduce inappropriate testing and misinterpretation. Future work should hence extend beyond assay performance to include implementation strategies, patient-centered outcomes, and health-system impact, ensuring that advances in heparin monitoring translate into safer and more consistent care across clinical settings.

## Figures and Tables

**Table 1:** Comparison of UFH and LMWH.

	UFH	LMWH
Mechanism	Binds antithrombin to inactivate factors IIa (thrombin) and Xa (more balanced anti-IIa activity).	Antithrombin-mediated inhibition predominantly of factor Xa (less anti-factor IIa due to shorter chains).
Pharmacokinetics	High protein/endothelial binding leading to nonlinear, variable kinetics with ~30% bioavailability.	Less non-specific binding leading to more predictable PK/PD with ~90–100% bioavailability (enoxaparin), ~80–90% (dalteparin).
Half life	0.5–1 h.	3–7 h.
Benefits	Rapid on/off, reversible.	Enables fixed or weight-adjusted SC dosing.
Dosing (Prophylaxis)	SC: 2500 IU or 5000 IU BID–TID (or weight-adjusted).	SC: 40 mg once daily or 0.5 mg/kg once daily.
Dosing (Therapeutic)	IV weight-based nomogram: ~80 U/kg bolus, then 18 U/kg/h infusion with titration (APTT or anti-Xa).	SC: 1 mg/kg BID or 1.5 mg/kg once daily. Routine labs not required in most adults; consider anti-Xa monitoring in pregnancy, extremes of body weight (<60 kg, >150 kg or BMI > 40 kg/m^2^), and renal impairment (CrCl < 30 mL/min).
Contraindications/Cautions	Use caution with bleeding diathesis, uncontrolled hypertension, recent gastrointestinal bleed, diabetic retinopathy; renal impairment requires caution and close monitoring.	Same cautions. Renal impairment: most guidelines suggest contraindicated when CrCl < 10 mL/min. Avoid benzyl alcohol-containing formulations in neonates; caution in pregnancy unless preservative-free.

APTT, activated partial thromboplastin time; BID (Bis in die): twice a day; BMI, body mass index; CrCl, creatinine clearance; IV, intravenous; LMWH, low-molecular-weight heparin; PD, pharmacodynamics; PK, pharmacokinetics; SC, subcutaneous; TID (Ter in die): three times a day; UFH, unfractionated heparin.

**Table 3:** Dosing strategies of LMWH in extremes of weight.

Weight	Management
BMI ≥ 40 kg/m^2^ or weight > 120 kg	LMWH: Continue 1 mg/kg every 12 h using total body weight (without dose cap), and consider selective peak anti-Xa monitoring (3–5 h post-dose after the 3rd–4th dose) if body weight is extreme or if clinical concerns arise [78,119]. UFH: Use actual or total body weight-based dosing with close monitoring (anti-Xa or APTT-based) [129]
Underweight (e.g., ≤45–50 kg)	LMWH: Consider lower prophylactic dose (e.g., 20–30 mg daily) and standard weight-based therapeutic dosing with closer clinical surveillance; trough anti-Xa can be used selectively to detect accumulation [115].

**Table 4:** Causes of heparin resistance and management considerations.

Mechanism	Pathophysiology	Diagnostic Clues	Management Considerations
Antithrombin deficiency	Reduced AT-dependent UFH effect	Anti-Xa low despite high dose of heparin; low AT activity	AT concentrate or FFP; continue UFH or switch to direct thrombin inhibitor (DTI)
Acute-phase proteins (increasing heparin binding)	Neutralization by FVIII, fibrinogen, VWF	APTT discordant with anti-Xa; high levels of acute-phase reactants	Titrate UFH by anti-Xa
PF4 neutralization	PF4 binds heparin, reducing activity	High platelet counts; clinical context	Manage underlying condition; consider alternative agents
Andexanet alfa exposure	Alters Xa pathway; assay interference	Clinical context/recent DOAC reversal	Use APTT temporally; delay anti-Xa-based UFH titration
Pseudo-resistance	Line/phlebotomy issues; sampling errors	Inconsistent laboratory tests vs. clinical picture	Re-collection from fresh venipuncture site; check quality control

APTT, activated partial thromboplastin time; AT, antithrombin; DOAC, direct oral anticoagulant; FFP, fresh-frozen plasma; PF4, platelet factor 4; VWF, von Willebrand factor; UFH, unfractionated heparin.

## Data Availability

As this manuscript represents a narrative review or synthesis of existing literature, no new data were created.

## Abbreviations

The following abbreviations are used in this manuscript:

Anti-Xa	anti-factor Xa
ACS	acute coronary syndrome
ACT	activated clotting time
AF	atrial fibrillation
APS	antiphospholipid syndrome
APTT	activated partial thromboplastin time
CPB	cardiopulmonary bypass
CrCl	creatinine clearance
CNS	central nervous system
DIC	disseminated intravascular coagulation
DOACs	direct oral anticoagulants
DVT	deep vein thrombosis
ECMO	extracorporeal membrane oxygenation
HIT	heparin-induced thrombocytopenia
HITT	heparin-induced thrombocytopenia with thrombosis
FIIa	factor IIa
FXa	factor Xa
LA	lupus anticoagulant
LMWH	low-molecular-weight heparin
OR	odds ratios
PF4	platelet factor 4
PE	pulmonary embolism
TEG-H	thromboelastography-heparin
UFH	unfractionated heparin
VET	viscoelastic testing
VTE	venous thromboembolism
